# A transcriptome analysis identifies molecular effectors of unconjugated bilirubin in human neuroblastoma SH-SY5Y cells

**DOI:** 10.1186/1471-2164-10-543

**Published:** 2009-11-19

**Authors:** Raffaella Calligaris, Cristina Bellarosa, Rossana Foti, Paola Roncaglia, Pablo Giraudi, Helena Krmac, Claudio Tiribelli, Stefano Gustincich

**Affiliations:** 1 International School for Advanced Studies (SISSA), Sector of Neurobiology, AREA Science Park, SS 14, Km 163 5, Basovizza, 34012 Trieste, Italy; 2 The Giovanni Armenise-Harvard Foundation Laboratory, SS 14, Km 163 5, Basovizza, 34012 Trieste, Italy; 3 Centro Studi Fegato, AREA Science Park, SS 14, Km 163 5, Basovizza, 34012 Trieste, Italy; 4 Universita' degli Studi di Trieste, 34012 Trieste, Italy

## Abstract

**Background:**

The deposition of unconjugated bilirubin (UCB) in selected regions of the brain results in irreversible neuronal damage, or Bilirubin Encephalopathy (BE). Although UCB impairs a large number of cellular functions in other tissues, the basic mechanisms of neurotoxicity have not yet been fully clarified. While cells can accumulate UCB by passive diffusion, cell protection may involve multiple mechanisms including the extrusion of the pigment as well as pro-survival homeostatic responses that are still unknown.

**Results:**

Transcriptome changes induced by UCB exposure in SH-SY5Y neuroblastoma cell line were examined by high density oligonucleotide microarrays. Two-hundred and thirty genes were induced after 24 hours. A Gene Ontology (GO) analysis showed that at least 50 genes were directly involved in the endoplasmic reticulum (ER) stress response. Validation of selected ER stress genes is shown by quantitative RT-PCR. Analysis of *XBP1 *splicing and DDIT3/CHOP subcellular localization is presented.

**Conclusion:**

These results show for the first time that UCB exposure induces ER stress response as major intracellular homeostasis in surviving neuroblastoma cells in vitro.

## Background

The toxic effects of bilirubin, a bile pigment produced during the catabolism of heme-containing compounds [1], have been documented in various biological systems. Nearly all newborn infants develop increased levels of unconjugated bilirubin (UCB) in the blood stream, clinically evident as neonatal jaundice [2]. Although this is a benign and transient phenomenon, abnormal accumulation of bilirubin may cause bilirubin encephalopathy ranging from minimal neurological injury to severe and permanent condition leading to neurodevelopmental dysfunctions [3]. Furthermore, infants affected by the genetic pathology of Crigler-Najjar I present high UCB plasma levels and are particularly exposed to bilirubin encephalopathy (BE) [4].

The basic mechanisms of hyperbilirubinemia neurotoxicity have not been fully clarified, although it has been shown that UCB may impair a large number of cellular functions, including energy metabolism [5], cell proliferation [6], DNA and protein synthesis [7], receptor functionality [8], and neurotransmitter uptake and release [9]. Furthermore, in the brain UCB seems more injurious to neurons than to glial cells, with effects that depend on the cellular subtype [10].

The endoplasmic reticulum (ER) plays a crucial role in the synthesis and folding of newly secretory and membrane proteins [11]. Any perturbation that compromises protein folding and functionality of the ER is referred to as ER stress. The ER stress response can promote cellular repair and survival by reducing the load of unfolded proteins through global attenuation of protein synthesis and by upregulating chaperones [12]. This response is collectively termed as the unfolded protein response (UPR) and is mediated by three ER transmembrane receptors: pancreatic ER kinase PERK (also known as eukaryotic translation initiation factor 2α (eIF2α) kinase 3 (EIF2AK3)), inositol-requiring enzyme 1 (IRE1) and activating transcription factor 6 (ATF6).

In resting cells, all of these ER stress receptors are maintained in an active state through their association with the ER chaperone GRP78 (also called BiP). An accumulation of unfolded proteins causes dissociation of GRP78 from PERK, IRE1 and ATF6, thereby initiating the UPR. Thus UPR is a pro-survival response to reduce the accumulation of unfolded proteins and restore normal function [13]. However, when misfolded-protein aggregation persists and ER stress cannot be resolved, signalling switches from a pro-survival to a pro-apoptotic pathway.

Recently, the ER has attracted attention as a key subcellular compartment in which the effects of several cellular stresses may contribute to pathological processes culminating in neuronal injury and degeneration. A central role of ER dysfunction is evident in various pathologies of the brain, including acute injuries (transient ischemia, trauma) and neurodegenerative disorders (Alzheimer's, Parkinson's and Huntington's diseases) [14].

The present study was designed to analyze the consequences of bilirubin exposure to better understand the molecular processes underlying neuronal damage and homeostasis. We used high density oligonucleotide microarrays to analyze the gene expression profile of human neuroblastoma SH-SY5Y cells upon UCB treatment. Gene expression data and experimental validation point to ER stress as the major intracellular homeostatic response in surviving cells.

## Results

### Characterization of the cell viability of UCB treated SH-SY5Y cells

**Figure 1:**
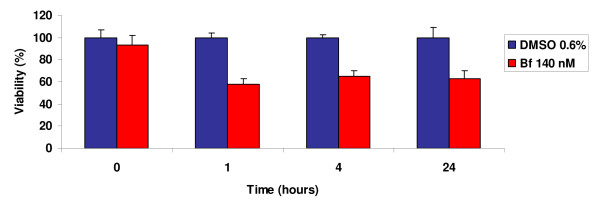
**Cell viability in SH-SY5Y cells treated with UCB**. SHSY5Y were exposed to UCB treatment at Bf 140 nM during a time course of 1, 4 and 24 hours and cell viability was evaluated by MTT assay. Cell viability of UCB-treated samples was expressed as a percentage of the respective samples treated with DMSO 0.6%. Data are expressed as the mean ± SD of 3 independent experiments performed in triplicate.

The human neuroblastoma SH-SY5Y cell line is a well-established *in vitro *cellular model to study the biological consequences of neuroactive molecules, drugs and toxins. To assess UCB effects, we have taken advantage of previous studies where the outcome of clinically relevant UCB concentrations was tested and the cytotoxic effects revealed [15,16]. The sensitivity of SH-SY5Y cells to UCB treatment was thus evaluated by measuring cell viability after exposing cells to a concentration of free bilirubin of 140 nM (Bf 140 nM) for 1, 4 and 24 h. As shown in Figure 1, cell viability was reduced to 60% already after 1 h of UCB treatment and the extent of reduction in cell viability never exceeded the 40% of the cell population, even with longer treatments.

### Gene expression analysis by oligonucleotide arrays in UCB treated SH-SY5Y cells

To examine the molecular events associated with the homeostatic response to UCB treatment, high density oligonucleotide microarrays (Affymetrix GeneChip Human U133A 2.0) were used to interrogate the expression of over 14,500 transcripts. RNA was isolated from SH-SY5Y neuroblastoma cells treated with Bf 140 nM for 1, 4 and 24 h. Experiments were performed in three biological replicates with control samples monitoring the effects of DMSO used as Bf solvent.

Analysis of the microarray hybridization data, as reported in Methods, produced a list of 258 probe sets with a false discovery rate (FDR) of 10%. This list corresponded to 230 genes upregulated after 24 h of UCB treatment ("late response"). No down-regulated genes were detected. The fold change of transcript levels ranged from 1.2 to 6.9, the majority of which being toward the lower end. A list of all differentially expressed genes is provided in the Additional file 1. No significant changes of gene expression were observed after 1 and 4 h of UCB treatment ("early response"). We obtained a list of 58 probe sets when a FDR of 0% was applied and a list of 196 probe sets with a FDR of 5%. Furthermore, 400 probe sets were induced with a FDR of 20% with no down-regulated genes (*data not shown*).

Differentially expressed genes were then examined for their Gene Ontology (GO) annotations concerning their cellular localization. As shown in Table 1 and as detailed in the Additional file 2, the analysis revealed that these genes were mainly distributed in the endoplasmic reticulum (ER), the Golgi apparatus (Golgi) and in the nucleus. To obtain insights into the pathways in which they were mainly involved, genes were sorted into distinct functional subgroups. UCB treatment induced genes belonging to functional categories related to the UPR in the protein secretory pathway (Table 2) as well as to regulation of transcription and DNA-dependent processes (Table 3). A set of up-regulated genes included plasma membrane transporters of the family of the solute carrier transporters (SLCs) and other genes involved in amino acid metabolism (Table 4). In the remaining part of the manuscript the attention will be focused on the UPR and ER stress responses, since the majority of up-regulated genes are well known members of these homeostatic pathways.

**Table 1:** Identification of the cellular localization of differentially expressed genes in response to 24 h of UCB treatment at Bf 140 nM in SH-SY5Y cells.

Localization	Number of genes	%
Endoplasmic reticulum	47	25.54

Golgi apparatus	25	13.59

Nucleus	41	22.28

The table shows the number and percentage (%) of the 217 total genes that were recognized as valid gene names in the gene_association.goa_human_hgnc file according to GO slim terms from the cellular ontology in regard to Endoplasmic reticulum, Golgi apparatus and nucleus localization. All details are reported in Table S2 in the Additional file 2.

**Table 2:** Differentially expressed genes involved in ER stress signalling and UPR in response to UCB treatment in SH-SY5Y cells.

Probe_Id	Gene_Name	Gene_Symbol	FC	Location
				

	**Stress sensing and signalling**			

218696_at	eukaryotic translation initiation factor 2-alpha kinase 3	*EIF2AK3*	2.2	ER membrane

				

	**Folding (Chaperones and oxidoreductase)**			

200068_s_at	Calnexin	*CANX*	1.5	ER membrane; cell surface

202843_at	DnaJ (Hsp40) homolog, subfamily B, member 9	*DNAJB9*	4.4	ER lumen

202842_s_at	DnaJ (Hsp40) homolog, subfamily B, member 9	*DNAJB9*	4.0	ER lumen

221782_at	DnaJ (Hsp40) homolog, subfamily C, member 10	*DNAJC10*	1.8	ER lumen

220012_at	ERO1-like beta (S. cerevisiae)	*ERO1LB*	2.1	ER; ER membrane

210627_s_at	glucosidase I	*GCS1*	1.5	ER lumen

200599_s_at	heat shock protein 90 kDa beta (Grp94), member 1	*HSP90B1*	1.5	ER lumen; cell surface

211936_at	heat shock 70 kDa protein 5 (glucose-regulated protein, 78 kDa)	*HSPA5*	2.3	ER lumen; cell surface

200825_s_at	hypoxia up-regulated 1	*HYOU1*	2.2	ER; ER lumen

203857_s_at	protein disulfide isomerase family A, member 5	*PDIA5*	1.7	ER; ER lumen

208639_x_at	protein disulfide isomerase family A, member 6	*PDIA6*	1.5	ER; ER lumen

207668_x_at	protein disulfide isomerase family A, member 6	*PDIA6*	1.4	ER; ER lumen

217716_s_at	Sec61 alpha 1 subunit (S. cerevisiae)	*SEC61A1*	1.6	ER membrane

219499_at	Sec61 alpha 2 subunit (S. cerevisiae)	*SEC61A2*	1.4	ER membrane

201916_s_at	SEC63 homolog (S. cerevisiae)	*SEC63*	1.5	ER membrane

				

	**Vesicle-mediated transport**			

202710_at	BET1 homolog (S. cerevisiae)	*BET1*	1.6	ER; Golgi;membrane

204017_at	KDEL (Lys-Asp-Glu-Leu) endoplasmic reticulum protein retention receptor 3/ERD2L3	*KDELR3*	1.6	ER; ER membrane

207264_at	KDEL (Lys-Asp-Glu-Leu) endoplasmic reticulum protein retention receptor 3	*KDELR3*	1.3	ER; ER membrane

212245_at	multiple coagulation factor deficiency 2	*MCFD2*	1.5	ER-Golgi intermediate compartment; Golgi; ER

215548_s_at	sec1 family domain containing 1	*SCFD1*	1.5	Golgi; ER; ER membrane; nucleus

201583_s_at	Sec23 homolog B (S. cerevisiae)	*SEC23B*	1.8	COPII vesicle coat; ER; Golgi

210293_s_at	Sec23 homolog B (S. cerevisiae)	*SEC23B*	1.6	COPII vesicle coat; ER; Golgi

202375_at	SEC24 related gene family, member D (S. cerevisiae)	*SEC24D*	2.1	COPII vesicle coat; ER; Golgi

215209_at	SEC24 related gene family, member D (S. cerevisiae)	*SEC24D*	1.6	COPII vesicle coat; ER; Golgi

200945_s_at	SEC31 homolog A (S. cerevisiae)	*SEC31A*	1.6	COPII vesicle coat; ER

200970_s_at	stress-associated endoplasmic reticulum protein 1	*SERP1*	1.9	ER; membrane

200971_s_at	stress-associated endoplasmic reticulum protein 1	*SERP1*	1.7	ER; membrane

200891_s_at	signal sequence receptor, alpha (translocon-associated protein alpha)	*SSR1*	1.6	ER; membrane

200087_s_at	transmembrane emp24 domain trafficking protein 2	*TMED2*	1.4	Golgi; ER-Golgi intermediate compartment

208757_at	transmembrane emp24 protein transport domain containing 9	*TMED9*	1.6	ER; ER membrane

212352_s_at	transmembrane emp24-like trafficking protein 10 (yeast)	*TMED10*	1.4	Golgi; ER-Golgi intermediate compartment

201398_s_at	translocation associated membrane protein 1	*TRAM1*	1.8	ER; membrane

				

	**Degradation**			

218333_at	Der1-like domain family, member 2	*DERL2*	1.6	ER membrane

203279_at	ER degradation enhancer, mannosidase alpha-like 1	*EDEM1*	2.1	ER lumen membrane

217168_s_at	homocysteine-inducible, endoplasmic reticulum stress-inducible, ubiquitin-like domain member 1	*HERPUD1*	4.1	ER membrane

202061_s_at	sel-1 suppressor of lin-12-like (C. elegans)	*SEL1L*	2.1	ER membrane

Genes are sub-categorized according to their GO Biological Process annotation. Fold changes (FC) are fold differences for cells treated with Bf 140 nM versus cells grown in DMSO 0.6% (controls) for 24 h. Affymetrix Probe Set-IDs, gene names and symbol, fold change (FC), and cellular location are indicated.

**Table 3:** Differential expression of genes mapping to "regulation of transcription, DNA-dependent" (GO Biological Process) in response to UCB treatment in SH-SY5Y cells.

Probe_Id	Gene_Name	Gene_Symbol	FC	Location
202672_s_at	activating transcription factor 3	*ATF3*	4.9	nucleus; nucleolus

200779_at	activating transcription factor 4 (tax-responsive enhancer element B67)	*ATF4*	1.7	nucleus; cytoplasm; plasma membrane

204998_s_at	activating transcription factor 5	*ATF5*	1.6	nucleus; cytoplasm

204999_s_at	activating transcription factor 5	*ATF5*	1.5	nucleus; cytoplasm

212501_at	CCAAT/enhancer binding protein (C/EBP), beta	*CEBPB*	4.9	nucleus; cytoplasm

204203_at	CCAAT/enhancer binding protein (C/EBP), gamma	*CEBPG*	2.4	Nucleus

212345_s_at	cAMP responsive element binding protein 3-like 2	*CREB3L2*	1.7	ER; ER membrane; integral to membrane; nucleus

207630_s_at	cAMP responsive element modulator	*CREM*	3.1	Golgi apparatus; cytoplasm; nucleus; transcription factor complex

209967_s_at	cAMP responsive element modulator	*CREM*	2.9	Golgi apparatus; cytoplasm; nucleus; transcription factor complex

214508_x_at	cAMP responsive element modulator	*CREM*	2.7	Golgi apparatus; cytoplasm; nucleus; transcription factor complex

209674_at	cryptochrome 1 (photolyase-like)	*CRY1*	1.4	Cytoplasm; mitochondrion; nucleus

209383_at	DNA-damage-inducible transcript 3	*DDIT3*	6.7	Nucleus

219551_at	ELL associated factor 2	*EAF2*	1.4	nucleus; nuclear speck

208436_s_at	interferon regulatory factor 7	*IRF7*	1.7	Cytoplasm; nucleus

201466_s_at	jun oncogene	*JUN*	3.5	cytosol; nuclear chromosome; nucleus; TF complex

201464_x_at	jun oncogene	*JUN*	2.8	cytosol; nuclear chromosome; nucleus; TF complex

203752_s_at	jun D proto-oncogene	*JUND*	1.6	Chromatin; nucleus

208961_s_at	Kruppel-like factor 6	*KLF6*	1.8	cellular component; cytoplasm; intracellular; nucleus

204970_s_at	v-maf musculoaponeurotic fibrosarcoma oncogene homolog G (avian)	*MAFG*	1.5	Nucleus

213696_s_at	mediator of RNA polymerase II transcription, subunit 8 homolog (S. cerevisiae)	*MED8*	1.6	mediator complex; nucleus

212803_at	NGFI-A binding protein 2 (EGR1 binding protein 2)	*NAB2*	1.5	Nucleus

203574_at	nuclear factor, interleukin 3 regulated	*NFIL3*	2.4	Nucleus

221803_s_at	nuclear receptor binding factor 2	*NRBF2*	1.3	Cytoplasm; nucleus

202861_at	period homolog 1 (Drosophila)	*PER1*	1.7	Cytoplasm; nucleus

36829_at	period homolog 1 (Drosophila)	*PER1*	1.6	Cytoplasm; nucleus

217861_s_at	prolactin regulatory element binding	*PREB*	1.9	ER; membrane; nucleus

202148_s_at	pyrroline-5-carboxylate reductase 1	*PYCR1*	1.5	mitochondrion

215670_s_at	SCAN domain containing 2	*SCAND2*	1.4	Nucleus

201471_s_at	sequestosome 1	*SQSTM1*	2.4	cytoplasm; cytosol; late endosome; nucleus

208991_at	signal transducer and activator of transcription 3 (acute-phase response factor)	*STAT3*	1.4	Cytoplasm; nucleus

213024_at	TATA element modulatory factor 1	*TMF1*	1.8	Golgi apparatus

218145_at	tribbles homolog 3 (Drosophila)	*TRIB3*	6.7	Nucleus

208763_s_at	TSC22 domain family, member 3	*TSC22D3*	1.6	Nucleus

218012_at	TSPY-like 2	*TSPYL2*	1.9	Cytoplasm; nucleolus; nucleus

200670_at	X-box binding protein 1	*XBP1*	2.6	Nucleus

207219_at	zinc finger protein 643	*ZNF643*	1.4	intracellular; membrane; nucleus

Fold changes (FC) are fold differences for cells treated with Bf 140 nM versus cells grown in DMSO 0.6% (controls) for 24 h. Affymetrix Probe Set-IDs, gene names and symbol, fold change (FC), and cellular location are indicated.

**Table 4:** Differential expression of genes involved in protein synthesis/translation, amino acid metabolism/transport and autophagy in response to UCB treatment in SH-SY5Y cells.

Probe_Id	Gene_Name	Gene_Symbol	FC	Location	Function
	**Protein synthesis/translation**				

201000_at	alanyl-tRNA synthetase	*AARS*	1.7	cytoplasm	tRNA aminoacylation

202402_s_at	cysteinyl-tRNA synthetase	*CARS*	1.8	cytoplasm	tRNA aminoacylation

202021_x_at	eukaryotic translation initiation factor 1	*EIF1*	1.4	cytoplasm	translation initiation factory activity

208290_s_at	eukaryotic translation initiation factor 5	*EIF5*	1.6	cytoplasm	translation initiation factory activity

208708_x_at	eukaryotic translation initiation factor 5	*EIF5*	1.5	cytoplasm	translation initiation factory activity

208693_s_at	glycyl-tRNA synthetase	*GARS*	1.8	cytoplasm	tRNA aminoacylation

213671_s_at	methionyl-tRNA synthetase	*MARS*	1.5	cytoplasm	tRNA aminoacylation

201475_x_at	methionyl-tRNA synthetase	*MARS*	1.5	cytoplasm	tRNA aminoacylation

213672_at	methionyl-tRNA synthetase	*MARS*	1.4	cytoplasm	tRNA aminoacylation

200802_at	seryl-tRNA synthetase	*SARS*	2.2	cytoplasm	tRNA aminoacylation

200629_at	tryptophanyl-tRNA synthetase	*WARS*	2.9	cytoplasm	tRNA aminoacylation

200628_s_at	tryptophanyl-tRNA synthetase	*WARS*	2.5	cytoplasm	tRNA aminoacylation

					

	**mTOR pathway and autophagy**				

217967_s_at	family with sequence similarity 129, member A	*FAM129A*	6.9	cytoplasm	not defined

217966_s_at	family with sequence similarity 129, member A	*FAM129A*	4.0	cytoplasm	not defined

202887_s_at	DNA-damage-inducible transcript 4	*DDIT4*	4.4	cytoplasm	not defined

208869_s_at	GABA(A) receptor-associated protein like 1	*GABARAPL1*	1.9	Golgi; ER; cytoplasm	protein binding

211458_s_at	GABA(A) receptors associated protein like 3	*GABARAPL3*	2.3	microtubule	protein binding

203827_at	WD repeat domain, phosphoinositide interacting 1	*WIPI1*	2.8	Golgi; autophagic vacuole membrane; cytoplasm	receptor binding

213836_s_at	WD repeat domain, phosphoinositide interacting 1	*WIPI1*	2.7	Golgi; autophagic vacuole membrane; cytoplasm	receptor binding

					

	**Amino acid metabolism/transport**				

217678_at	solute carrier family 7, (cationic amino acid transporter, y+ system) member 11	*SLC7A11*	6.8	membrane	amino acid transport

209921_at	solute carrier family 7, (cationic amino acid transporter, y+ system) member 11	*SLC7A11*	6.6	membrane	amino acid transport

201195_s_at	solute carrier family 7 (cationic amino acid transporter, y+ system), member 5	*SLC7A5*	3.2	membrane	amino acid transport

200924_s_at	solute carrier family 3 (activators of dibasic and neutral amino acid transport), member 2	*SLC3A2*	3.8	membrane	amino acid transport

209610_s_at	solute carrier family 1 (glutamate/neutral amino acid transporter), member 4	*SLC1A4*	2.3	membrane	amino acid transport

212810_s_at	solute carrier family 1 (glutamate/neutral amino acid transporter), member 4	*SLC1A4*	2.1	membrane	amino acid transport

212811_x_at	solute carrier family 1 (glutamate/neutral amino acid transporter), member 4	*SLC1A4*	2.1	membrane	amino acid transport

212110_at	solute carrier family 39 (zinc transporter), member 14	*SLC39A14*	1.6	membrane	zinc ion transport

203165_s_at	solute carrier family 33 (acetyl-CoA transporter), member 1	*SLC33A1*	2.2	ER; membrane	acetyl-CoA transporter activity

203164_at	solute carrier family 33 (acetyl-CoA transporter), member 1	*SLC33A1*	1.9	ER; membrane	acetyl-CoA transporter activity

202433_at	solute carrier family 35, member B1	*SLC35B1*	1.6	ER; membrane	carbohydrate transport

221024_s_at	solute carrier family 2 (facilitated glucose transporter), member 10	*SLC2A10*	1.6	membrane	glucose transport

Genes are sub-categorized according to their GO Biological Process annotation. Fold changes (FC) are fold differences for cells treated with Bf 140 nM versus cells grown in DMSO 0.6% (controls) for 24 h. Affymetrix Probe Set-IDs, gene names and symbol, fold change (FC), cellular location are indicated.

### UCB treatment induced expression of ER stress genes in the UPR of the secretory pathway

UPR serves many aspects of the secretory pathway and is predominantly involved in the response to stress conditions when proteins are misfolded. GO categories of UPR components induced by UCB are shown in Table 2. These include stress sensing and signalling, protein folding, translocation, vesicular transport and ER-associated degradation. Genes known or predicted by sequence homology to play a physiological role in secretion or in the biogenesis of organelles were also increased.

The largest group of up-regulated genes was represented by ER-resident molecular chaperone and oxido-reductase proteins. Among the molecular chaperones, we found established UPR target genes such as *HSPA5 *(heat shock 70 kDa protein 5) (upregulated 2.3 times), known also as GRP78 (glucose-regulated protein, 78 kDa) or BiP [17], *HSP90B *(heat shock protein 90 kDa beta (Grp94) (1.5), member 1), *DNAJB9 *(4.4) and *DNAJC10 *(1.8)[18]. Additional genes included *PDIA5 *(1.7), *PDIA6 *(1.5) and *ERO1LB *(2.1) that are involved in oxidative protein folding [19].

A second major group was composed by genes involved in co-translational translocation of proteins across the ER membrane, to the Golgi and in the anterograde/retrograde transport back into the cytosol. The group included the signal sequence receptor *SSR1 *(1.6), the translocating chain-associating membrane protein *TRAM1 *(1.8), members of the SEC61 complex, such as *SEC61A1 *(1.6), *SEC61A2 *(1.4) and *SEC63 *(1.5), and *SERP1 *(Stress-associated Endoplasmic Reticulum Protein 1) (1.9), also known as RAMP4 (Ribosome-Associated Membrane Protein 4), that is a Sec61-associated polypeptide specifically induced by ER stress [20]. Up-regulated genes were also represented by the coated vesicle membrane proteins, such as *SEC23B *(1.8), *SEC24D *(2.1), *SEC31A *(1.6) and *SCFD1 *(1.5), as well as by the transmembrane trafficking proteins *TMED2 *(1.4), *TMED9 *(1.6) and *TMED10 *(1.4).

The ER-associated degradation (ERAD) system eliminates misfolded proteins by their retrotranslocation across the ER membrane into the cytosol, where ubiquitin-conjugating enzymes target them for proteosomal degradation. ERAD requires a number of dedicated ER-resident factors. Indeed, *HERPUD1 *or HERP (Homocysteine-induced ER Protein) (4.1) [21] was significantly induced as well as *EDEM1 *(ER Degradation Enhancer Mannosidase alpha-like 1) (2.1) [22], *SEL1L *(2.1), a transmembrane cofactor of the E3 ubiquitin ligase HRD1 [23], and *DERL2 *(Derlin membrane-like domain family member 2) (1.6) whose product associates with EDEM1 and is a specific target of the IRE1 branch of the UPR response [24].

### UCB treatment induced expression of proteins relevant in the regulation of transcription during ER stress response

As shown in Table 3, several differentially expressed genes were classified into the GO category of "regulation of transcription, DNA-dependent". Up-regulated transcription factors included two established UPR target genes: *DDIT3 *(6.7), also known as CHOP or C/EBP homologous protein, and *XBP1*, X box-binding protein 1 (2.6). CHOP is a 29-kDa leucine zipper transcription factor that is ubiquitously expressed at a low level and robustly up-regulated in response to various stress conditions [25]. CHOP is proapoptotic and a key mediator of ER stress-induced cell death [26,27]. *XBP1 *gene, after a nonconventional splicing event [28], codes for a transcription activator that regulates a subset of ER-resident chaperones that are essential for protein folding, maturation and degradation in the ER [29]. Additional stress-responsive transcriptional regulators induced by UCB included *ATF3 *(4.9), *ATF4 *(1.7), *ATF5 *(1.6), *CEBPB *(4.9), *CEBPG *(2.4), *JUN *(3.5) and *JUND *(1.6). Interestingly, although *ATF4 *showed a moderate induction, several of its downstream targets were strongly up-regulated, e.g. *DDIT3*, *ATF3*, *CEBPB*, *CEBPG*, *CREB3L2 *(1.7) and *TRIB3 *(6.7). *TRIB3 *(also known as NIPK, SKIP3, TRB3 and SINK) is an ER stress-inducible gene that is involved in CHOP-dependent cell death as a second messenger during ER stress [30].

### UCB treatment induced expression of genes involved in protein synthesis/translation, autophagy and aminoacid transport/metabolism

The translational machinery for a new boost of protein synthesis has been suggested to be amplified during UPR-associated translational attenuation [13,31]. As shown in Table 4, we observed an up-regulation of 6 tRNA synthetase genes, such as *AARS *(1.7), *CARS *(1.8), *GARS *(1.8), *MARS *(1.5), *SARS *(2.2) and *WARS *(2.9), which are involved in tRNA processing. We also observed the induction of 2 eukaryotic translation initiation factors, that are *EIF1 *(1.4) and *EIF5 *(1.6). One of the stronger up-regulated genes was *FAM129A *(family with sequence similarity 129, member A) (6.9), also known as NIBAN, which has been recently demonstrated to be involved in the ER stress response and to positively affect the protein translation machinery by regulating the mTOR pathway [32]. Interestingly, the list of genes induced during UCB treatment also includes *DDIT4 *(DNA-Damage-Inducible Transcript 4) (4.4), also known as REDD1/RTP801/Dig2, that is another well-known regulator of the mTOR pathway [33]. The mTOR-dependent pathway is strongly connected to the autophagic machinery, and indeed autophagy-related genes such as *GABARAPL1 *(GABA(A) receptor-associated protein like 1 or ATG8) (1.9), *GABARAPL3 *(GABA(A) receptors-associated protein like 3 (pseudogene)) (2.3) and *WIPI1 *(WD repeat domain, phosphoinositide interacting 1 gene or Atg18) (2.8) were also positively differentially expressed, suggesting that surviving neuroblastoma cells may present an induction of autophagy [34,35].

UCB exposure also activated genes involved in amino acid metabolism (Table 4). Among the strongest up-regulated genes, *SLC7A11 *(6.8) and *SLC3A2 *(3.8) were identified. These two genes encode respectively for xCT, the light chain subunit, and for 4F2hC, the glycosylated heavy-chain subunit, that are components of the system xCT, anionic amino acid transport system specific for cystine and glutamate [36]. Recently, the UCB-induced SLC7A5 (3.2) has been shown to physically and functionally interact with SLC3A2, forming a bidirectional amino acid transporter and representing a key regulator of both the mTOR pathway and autophagy [37].

### Validation of selected genes by quantitative RT-PCR (qRT-PCR)

**Figure 2:**
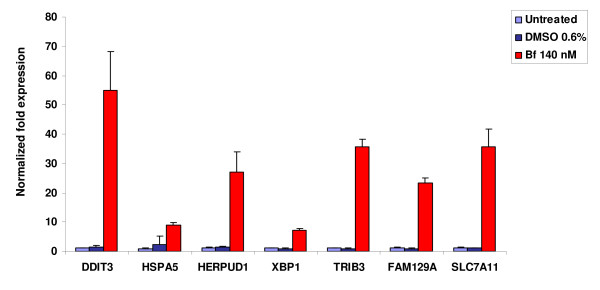
**Analysis of gene expression related to the ER stress response**. Gene expression levels of selected genes (X axis) were analyzed by qRT-PCR. Y axis represents the gene expression level normalized to housekeeping genes (*GAPDH *and *β-actin*) of SH-SY5Y cells not treated (untreated) or treated with vehicle (DMSO 0.6%) or UCB (Bf 140 nM) for 24 hours. These results represent at least three RNA samples per experimental condition run in triplicate.

For validation, we selected genes considered of interest based on their involvement in ER stress response. Gene expression levels of *DDIT3*, *HSPA5*, *XBP1*, *HERPUD1*, *TRIB3*, *FAM129A *and *SLC7A11 *were verified by qRT-PCR. Results are reported in Figure 2. The fold differences determined by qRT-PCR were very similar to those obtained by the high density oligonucleotide microarray gene expression analysis.

### Analysis of *XBP1 *splicing and DDIT3/CHOP subcellular localization in UCB treated SH-SY5Y cells

**Figure 3:**
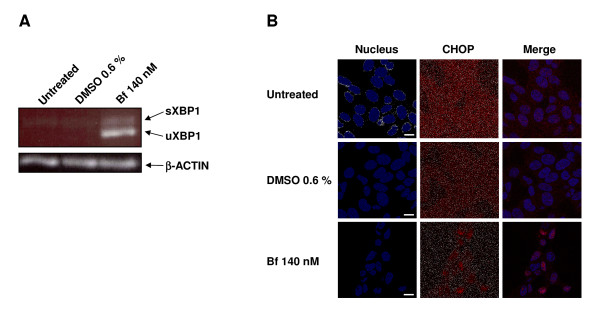
**Analysis of *XBP1 *splicing and DDIT3/CHOP subcellular localization in SH-SY5Y cells treated with UCB**. (A) mRNA levels of the spliced form of *XBP1 *(s - spliced, u - unspliced), indicator of ER stress, were examined by RT-PCR after 24 h of treatment. The amplified products were separated on a 2% agarose gel and visualized with ethidium bromide staining. Data are representative of n ≥ 2 separate experiments per condition point. (B) The expression of CHOP (shown in red) was examined with immunocytochemistry as described in Methods. Representative images are shown for SH-SY5Y cells not treated (untreated) or treated with vehicle (DMSO 0.6%) or UCB (Bf 140 nM). The nuclei (shown in blue) were visualized with DAPI staining. Bar = 10 μm.

We investigated two independent events that are known activators of the ER stress transcriptional program: unconventional splicing of *XBP1 *and cytoplasm/nuclear shuttling of DDIT3/CHOP. The splicing of *XBP1 *transcript changes its open reading frame to code for a transcription factor (spliced XBP1 or sXBP1) that activates the expression of a subset of ER stress/UPR related genes [28,29]. Here we show that UCB treatment is indeed able to trigger this unconventional splicing (Figure 3A). The nuclear localization of the transcription factor DDIT3/CHOP [25] is one of the most commonly used indicators of ER stress. As presented in Figure 3B, we observed a diffuse cytoplasmic localization in untreated and DMSO 0.6% control cells, but a major nuclear staining in cells treated with Bf 140 nM. This indicates activation of a CHOP-dependent transcriptional program. These data represent an independent proof of the induction of a vigorous ER stress.

## Discussion

Bilirubin encephalopathy is one of the consequences of severe hyperbilirubinemia and is characterized by multifocal deposition of UCB in selected regions of the brain, resulting in temporary or permanent impairment of auditory, motor or mental functions [38]. Cellular UCB level is closely related to the free concentration (Bf) of the pigment in plasma and to the mechanisms regulating the traffic of UCB among plasma, cerebral spinal fluid and cells. Although UCB is formed in virtually all cells and may enter by passive diffusion [39], intracellular concentration is determined by cellular export and metabolic transformation of the pigment. Interestingly, only certain neuronal cell types present a UCB susceptibility to undergo necrosis and apoptosis [40], whereas astrocytes are relatively spared. This has been linked to a higher content of MRP1, a transporter with high affinity to UCB [41] able to keep the intracellular UCB concentration low. Importantly, the level of MRP1 expression in human neuroblastoma SH-SY5Y cells is inversely and linearly correlated with UCB toxic effects [16].

The molecular mechanisms of intracellular UCB toxicity are still unclear [42]. UCB has been initially proved to be a pro-apoptotic agent, suppressing cell growth by inducing DNA fragmentation, mitochondrial release of cytochrome c, activation of caspase-3 and cleavage of poly(ADP)ribose polymerase [40]. More recently, oxidative stress has emerged as a potential crucial event, since its generation mirrors UCB-mediated apoptosis [43,44]. In various cellular systems, UCB causes reactive oxygen species (ROS) production, protein oxidation, lipid peroxidation and disruption of glutathione metabolism [43,45-47]. Furthermore, UCB-mediated oxidative stress is also in part responsible for inhibition of cell growth [48,49]. Inhibition of cell proliferation has been indeed observed in primary vascular smooth muscle cells *in vitro *as well as inferred by gene expression data on the repression of cell cycle-related genes in the liver of a mouse model of Crigler-Najjar type I disease [50]. To study the effects of intracellular UCB on neurons, we took advantage of the human neuroblastoma SH-SY5Y cell line. These cells have been widely used in neurobiology as the *in vitro *system of choice to dissect molecular pathways leading to neurodegeneration. Previous studies have already shown that neuroblastoma cells are a good *in vitro *model to address the consequences of clinically relevant UCB concentrations [16]. Although we are aware of the limitations in the use of a neuroblastoma cell line to recapitulate dysfunctions that occur in neonatal human brain, this experimental model allowed us to dissect in details the molecular events elicited by UCB.

Treatment with Bf 140 nM was highly toxic, triggering death in 40% of the cells within one hour. This is expected since the accepted threshold for bilirubin toxicity approximately occurs at 70 nM [5]. 63% reduction in cell viability at 4 h after bilirubin exposure were previously demonstrated in the rat neuroblastoma N-115 cell line [7]. Furthermore, Silva RF *et al*. [51] showed 85 nM Bf triggered deleterious effects on mitochondrial function.

A full understanding of molecular mechanisms of cell death will require additional investigation. UCB has been previously proved to induce apoptosis in neuroblastoma cells, although necrosis could not be excluded [52].

Furthermore, the criteria to distinguish sensitive from insensitive neuroblastoma cells remain unclear. One may speculate that subtle heterogeneity in the level of differentiation may play a role in susceptibility to insults. Alternatively, cell death induction may depend on cell cycle position.

We then focused on the 60% of surviving cells to test the hypothesis that these neurons may express a homeostatic response.

A gene expression approach was carried out to identify gene expression patterns and signalling pathways induced upon UCB treatment. No significant changes of gene expression were observed after 1 and 4 h of UCB treatment. After 24 h, 230 genes were induced while no down-regulated genes were observed even when an FDR of 20% was applied.

The lack of an "early response" is surprising since it fails to provide a potential mechanism for cell survival within the first hour. Therefore, present data suggest that it is very unlikely that the molecular pathways induced at 24 hours play a role in the survival of cells after one hour of treatment.

GO analysis of the "late response" genes proved for the first time that UCB treatment induces an ER stress response in neuroblastoma cells *in vitro*. Evidences for ER stress involvement are overwhelming. Among others, genes induced by UCB treatment include several molecular chaperones like BiP, a well-known marker of ER overload, as well as molecular components of the ERAD system, that has been recently positioned in a central stage among the molecular mechanisms of neurodegeneration [53,54]. Furthermore, two well-known events in ER stress, such as *XBP1 *unconventional splicing and *DDIT3*/CHOP nuclear relocation, were proved to be triggered by UCB treatment, therefore providing additional experimental evidences.

UCB also altered several components of the translational machinery including tRNA synthesis genes and translation initiation factors. The translation machinery is considered one of the major targets of the ER stress response to regulate protein synthesis and decrease protein overload. Since no data are available in literature about the relationship between UCB intracellular concentration and translation rate, there is a compelling need for an accurate proteomic analysis of UCB effects in SH-SY5Y neuroblastoma cells. A complementary cellular strategy in ER stress conditions includes an increase in protein degradation. In this context some UCB-induced genes suggested a role for autophagy. It is of notice that autophagy has been recently associated with neurodegenerative diseases as well as with inhibition of cell growth and entering into a quiescent state [55,56]. This may correlate to previous reports showing a severe inhibition of cell proliferation mediated by UCB [49]. Further experimental validation is needed to prove an increase of autophagy.

Finally, the induction of both subunits of the transport system X(C)(-) was also observed: SLC3A2 (4F2hc) and SLC7A11 (xCT) are able to heterodimerize to mediate cystine-glutamate exchange and regulate intracellular glutathione levels. Since the maintenance of a high glutathione concentration may be protective [57], we speculate that induction of the X(C)(-) transport system may represent a major pro-survival response, as has been recently shown in neurons [58]. Interestingly, we could not observe any significant differential gene expression of *MRP1 *at any point of the time course suggesting that this transporter plays a negligible role in UCB-induced adaptation in this *in vitro *model.

## Conclusion

In summary, the present study shows for the first time that intracellular accumulation of UCB in surviving neuroblastoma SH-SY5Y cell line provokes ER stress.

## Methods

### Chemicals

UCB was purchased from Sigma (St. Louis, MO, USA) and purified as described by McDonagh and Assisi [59]. UCB was dissolved in 0.6% v/v of DMSO and diluted in 100 μL of complete medium supplemented with 15% Fetal Bovine Serum containing 54 μM albumin. Bf concentration was 140 nM, measured as described previously [60]. 3(4,5-dimethylthiazolyl-2)-2,5 diphenyl tetrazolium (MTT), HPLC-grade dimethyl sulfoxide (DMSO), and all other chemicals were purchased from Sigma, unless otherwise specified.

### Experimental treatment of SH-SY5Y cell line

SH-SY5Y human neuroblastoma cells (ATCC CRL-2266) were cultured in a humidified incubator at 37°C and 5% CO2 in F12-EMEM (1:1) containing 15% foetal bovine serum (FBS), 2 mM L-glutamine, 25 μg/mL gentamicin, 100 U/ml penicillin, and 100 μg/mL streptomycin at 37°C with 5% CO_2_. Cells were seeded at 6 × 10^6 ^cells in 75 cm^2 ^flasks and grown for 24 h (cells reached 70-80% confluence). The medium was then replaced with experimental medium, which was either free medium (untreated samples) or 140 nM Bf medium (Bf 140 nM samples), or 0.6% DMSO medium (DMSO 0.6% samples) and cells were allowed to grow. After 1, 4 and 24 h cells were collected and processed for cell viability (MTT cell viability assay), microarray, quantitative RT-PCR and immunofluorescence analyses. Separate flasks of cells were used for each of the treatments and assays. Experiments were performed at least in three independent biological replicates at all time points.

### MTT cell viability assay

Cells were treated as described above and the MTT cell viability assay was performed as previously described by Denizot and Lang [61]. Absorbance was measured at 562 nm using a microtiter plate reader (LC 400, Beckman Coulter, Milan, Italy). Cell viability of UCB treated samples was expressed as a percentage of the respective samples treated with DMSO alone.

### RNA extraction and quantitative RT-PCR (qRT-PCR)

Total RNA was isolated using the TRIzol reagent (Invitrogen) following the manufacturer's instructions. Single strand cDNA was obtained from 1 μg of purified RNA using the iSCRIPT™ cDNA Synthesis Kit (Bio-Rad) according to manufacturer's instructions. Quantitative RT-PCR was performed using SYBR-Green PCR Master Mix (Applied Biosystem) and an iCycler IQ Real Time PCR System (Bio-Rad). Sequence of gene specific primers used for qRT-PCR is provided in Table 5. Expression of the gene of interest was normalized to house keeping genes (glyceraldehyde-3-phosphate dehydrogenase or *GAPDH *and *β-actin*) and the initial amount of the template of each sample was determined as relative expression versus one of the samples chosen as reference. The relative expression of each sample was calculated by the formula 2 exp^-ΔΔCt ^(User Bulletin 2 of the ABI Prism 7700 Sequence Detection System). *GAPDH *and *β-actin *expressions are not modified under the present experimental conditions (data not shown). *XBP1 *splicing was studied using reverse transcriptase-polymerase chain reaction. cDNA was synthesized as herein reported. Human *XBP1 *primers (forward: 5'-AAACAGAGTAGCAGCTCAGACTGC-3'; reverse: 5'-TCCTTCTGGGTAGACCTCTGGG AG-3') were designed to generate cDNA products encompassing the IRE1 cleavage site as previously described [28]. The unspliced (uXBP1) and spliced (sXBP1) mRNAs generate 473- and 447-bp cDNA products, respectively. The amplified products were separated on a 2% agarose gel and visualized with ethidium bromide staining.

**Table 5:** Primer sequences used for qRT-PCR.

Gene name	Forward	Reverse
*DDIT3*	5'-CACTCTCCAGATTCCAGTCAG-3'	5'-AGCCGTTCATTCTCTTCAGC-3'

*HSPA5*	5'-GCACAGACAGATTGACCTATTG-3'	5'-GTAGCACAGGAGCAC-3'

*HERPUD1*	5'-CTAGATGGCGAGCAGACC-3'	5'-GAGTCAGGTGATCCAGTCC-3'

*XBP1*	5'-ATGGATTCTGGCGGTATTG-3'	5'-CTGGGTCCTTCTGGGTAG-3'

*TRIB3*	5'-CGTGATCTCAAGCTGTGTCG-3'	5'-GAGTCCTCCAGGTTCTCC-3'

*FAM129A*	5'-CCAGGAGTCAGAGGAAGAGAAG-3'	5'-GTTGCCACAGGATTCACCAC-3'

*SLC7A11*	5'-GGTGGTGTGTTTGCTGTC-3'	5'-GCTGGTAGAGGAGTGTGC-3'

*β-ACTIN*	5'-CGCCGCCAGCTCACCATG-3'	5'-CACGATGGAGGGGAAGACGG-3'

*GAPDH*	5'-TCTCTGCTCCTCCTGTTC-3'	5'-GCCCAATACGACCAAATCC-3'

### Microarray processing and data analysis

Total RNA was extracted as described above and purified using the RNeasy mini kit (Qiagen). The quality of total RNA was assessed using a bioanalyzer (Agilent 2100; Agilent Technologies) and RNA was quantified by using a ND-1000 Nanodrop spectrophotometer. Ten μg of each total RNA sample was labelled according to the standard one-cycle amplification and labelling protocol developed by Affymetrix (Santa Clara, CA). Labelled cRNA was hybridized on Affymetrix GeneChip Human U133A 2.0 Arrays containing over 14,500 transcripts. Hybridized GeneChips were stained, washed (GeneChip Fluidic Station 450) and scanned (GeneChip Scanner 3000 7G). Cell intensity values and probe detection calls were computed from the raw array data using the Affymetrix GeneChip Operating Software (GCOS). Further data processing was performed in the R computing environment http://www.r-project.org/, version R 2.5.0 for Windows) using packages from the BioConductor software project http://www.bioconductor.org/. Variance-stabilizing normalization was applied [62], using the "justvsn" function from the "vsn" library. Normalized data were then filtered based on the Affymetrix detection call, so that only probes that had a Present call in at least one of the arrays were retained [63]. Data were then imported in the MultiExperiment Viewer (MeV) software [64] (version 4.0.01 for Windows XP), and statistical analysis was performed with the SAM (Significance Analysis of Microarrays) module [65,66]. A False Discovery Rate (FDR) of about 10% (i.e. 9.317%) was applied to detect significantly differentially expressed genes. Microarray data have been deposited in the NCBI Gene Expression Omnibus (GEO) with Accession Number GSE16768. Differentially expressed genes were then specifically examined, based on their Gene Ontology annotation [67], for their cellular localization and for their involvement in some biological processes of interest. GO slim annotations were based on the Generic GO Term Mapper http://go.princeton.edu/cgi-bin/GOTermMapper using the Gene Association File goa_human_hgnc (Generic GO slim).

### Immunocytochemistry

For immunofluorescence experiments, SH-SY5Y cells were cultured on glass slides overnight, fixed in 4% paraformaldehyde for 10 minutes, washed with PBS two times, treated with 0.1 M glycine for 5 minutes in PBS and permeabilized with 0.1% Triton X-100 in PBS for additional 5 minutes. After washing with PBS and blocking with 0.2% BSA in PBS, cells were incubated with the anti-CHOP 1:100 (Santa Cruz Biotechnology) diluted in 0.2% BSA in PBS for 90 minutes at room temperature. After washing, cells were incubated with AlexaFluor 594 (Dako Cytomation)-labelled anti-mouse secondary antibody for 1 h. For nuclear staining, cells were incubated with 1 μg/mL DAPI for 5 minutes. Cells were washed and mounted with Vectashield mounting medium (Vector). Images were collected using a confocal microscope (LEICA TCS SP2).

## Authors' contributions

RC performed data analysis, assisted with the generation of microarray data, validated targets by qRT-PCR, wrote and revised the manuscript. CB and PG performed the cell culture and the MTT assays. PR performed the bioinformatic and statistical analysis of the microarray experiments. RF performed some qRT-PCR experiments. HK produced the microarray data at the CBM Genomics unit (Trieste, Italy). SG conceived, designed and wrote the paper. CT and SG provided support, direction and oversight of the experiments and revised the final manuscript. All authors read and approved the final version of the manuscript.

## Supplementary Material

Additional file 1 **Table S1:** Differentially expressed genes in response to 24 h of UCB treatment at Bf 140 nM in SH-SY5Y cells. Affymetrix Probe Set IDs, gene names and symbols and fold changes (FC) are indicated. Click here for file

Additional file 2 **Table S2**. Identification of the GO cellular components categories of differentially expressed genes in response to UCB-treatment in SH-SY5Y cells (GO slim annotation as reported in Table 1). Click here for file

## References

[B1] Ostrow JD Mukerjee P Tiribelli C Structure and binding of unconjugated bilirubin: relevance for physiological and pathophysiological function J Lipid Res 1994 35 1715 1737 7852850

[B2] Gourley GR Bilirubin metabolism and kernicterus Adv Pediatr 1997 44 173 229 9265971

[B3] Cashore WJ The neurotoxicity of bilirubin Clin Perinatol 1990 17 437 448 2196139

[B4] Seppen J Bosma PJ Goldhoorn BG Bakker CT Chowdhury JR Chowdhury NR Jansen PL Oude Elferink RP Discrimination between Crigler-Najjar type I and II by expression of mutant bilirubin uridine diphosphate-glucuronosyltransferase J Clin Invest 1994 94 2385 2391 10.1172/JCI117604 7989595 PMC330068

[B5] Roger C Koziel V Vert P Nehlig A Effects of bilirubin infusion on local cerebral glucose utilization in the immature rat Dev Brain Res 1993 76 115 130 10.1016/0165-3806(93)90129-X 8306423

[B6] Keshavan P Schwemberger SJ Smith DL Babcock GF Zucker SD Unconjugated bilirubin induces apoptosis in colon cancer cells by triggering mitochondrial depolarization Int J Cancer 2004 112 433 445 10.1002/ijc.20418 15382069

[B7] Kashiwamata S Aono S Semba RK Characteristic changes of cerebellar proteins associated with cerebellar hypoplasia in jaundiced Gunn rat and the prevention of these by phototherapy Experientia 1980 36 1143 1144 10.1007/BF01976089 7418786

[B8] Hoffman DJ Zanelli SA Kubin J Mishra OP Delivoria-Papadopoulos M The in vivo effect of bilirubin on the *N*-methyl-D-aspartate receptor/ion channel complex in the brains of newborn piglets Pediatr Res 1996 40 804 808 10.1203/00006450-199612000-00005 8947954

[B9] Ochoa EL Wennberg RP An Y Tandon T Takashima T Nguyen T Chui A Interactions of bilirubin with isolated presynaptic nerve terminals: functional effects on the uptake and release of neurotransmitters Cell Mol Neurobiol 1993 13 69 86 10.1007/BF00712990 8096165 PMC11566801

[B10] Notter MFD Kendig JW Differential sensitivity of neural cells to bilirubin toxicity Exp Neurol 1986 94 670 682 10.1016/0014-4886(86)90246-3 3780913

[B11] Ron D Walter P Signal integration in the endoplasmic reticulum unfolded protein response Nat Rev Mol Cell Biol 2007 8 519 529 10.1038/nrm2199 17565364

[B12] Kaufman RJ Orchestrating the unfolded protein response in health and disease J Clin Invest 2002 110 1389 98 12438434 10.1172/JCI16886 PMC151822

[B13] Schröder M Kaufman RJ The mammalian unfolded protein response Annu Rev Biochem 2005 74 739 89 10.1146/annurev.biochem.73.011303.074134 15952902

[B14] Scheper W Hoozemans JJ Endoplasmic reticulum protein quality control in neurodegenerative disease: the good, the bad and the therapy Curr Med Chem 2009 16 615 626 10.2174/092986709787458506 19199926

[B15] Calligaris SD Bellarosa C Giraudi P Wennberg RP Ostrow JD Tiribelli C Cytotoxicity is predicted by unbound and not total bilirubin concentration Pediatr Res 2007 62 576 580 10.1203/PDR.0b013e3181568c94 18049372

[B16] Corich L Aranda A Carrassa L Bellarosa C Ostrow JD Tiribelli C The cytotoxic effect of unconjugated bilirubin in human neuroblastoma SH-SY5Y cells is modulated by the expression level of MRP1 but not MDR1 Biochem J 2009 417 305 312 10.1042/BJ20080918 18713069

[B17] Bertolotti A Zhang Y Hendershot LM Harding HP Ron D Dynamic interaction of BiP and ER stress transducers in the unfolded-protein response Nat Cell Biol 2000 2 326 332 10.1038/35014014 10854322

[B18] Ushioda R Hoseki J Araki K Jansen G Thomas DY Nagata K ERdj5 is required as a disulfide reductase for degradation of misfolded proteins in the ER Science 2008 321 569 572 10.1126/science.1159293 18653895

[B19] Ellgaard L Helenius A Quality control in the endoplasmic reticulum Nat Rev Mol Cell Biol 2003 4 181 191 10.1038/nrm1052 12612637

[B20] Hori O Miyazaki M Tamatani T Ozawa K Takano K Okabe M Ikawa M Hartmann E Mai P Stern DM Kitao Y Ogawa S Deletion of SERP1/RAMP4, a component of the endoplasmic reticulum (ER) translocation sites, leads to ER stress Mol Cell Biol 2006 26 4257 4267 10.1128/MCB.02055-05 16705175 PMC1489087

[B21] Kokame K Agarwala KL Kato H Miyata T Herp, a new ubiquitin-like membrane protein induced by endoplasmic reticulum stress J Biol Chem 2000 275 32846 32853 10.1074/jbc.M002063200 10922362

[B22] Oda Y Hosokawa N Wada I Nagata K EDEM as an acceptor of terminally misfolded glycoproteins released from calnexin Science 2003 299 1394 1397 10.1126/science.1079181 12610305

[B23] Neuber O Jarosch E Volkwein C Walter J Sommer T Ubx2 links the Cdc48 complex to ER-associated protein degradation Nat Cell Biol 2005 7 993 998 10.1038/ncb1298 16179953

[B24] Oda Y Okada T Yoshida H Kaufman RJ Nagata K Mori K Derlin-2 and Derlin-3 are regulated by the mammalian unfolded protein response and are required for ER-associated degradation J Cell Biol 2006 172 383 393 10.1083/jcb.200507057 16449189 PMC2063648

[B25] Wang XZ Lawson B Brewer JW Zinszner H Sanjay A Mi LJ Boorstein R Kreibich G Hendershot LM Ron D Signals from the stressed endoplasmic reticulum induce C/EBP-homologous protein (CHOP/GADD153) Mol Cell Biol 1996 16 4273 4280 8754828 10.1128/mcb.16.8.4273 PMC231426

[B26] Friedman AD GADD153/CHOP, a DNA damage-inducible protein, reduced CAAT/enhancer binding protein activities and increased apoptosis in 32D c13 myeloid cells Cancer Res 1996 56 3250 3256 8764117

[B27] Oyadomari S Mori M Roles of CHOP/GADD153 in endoplasmic reticulum stress Cell Death Differ 2004 11 381 389 10.1038/sj.cdd.4401373 14685163

[B28] Calfon M Zeng H Urano F Till JH Hubbard SR Harding HP Clark SG Ron D IRE1 couples endoplasmic reticulum load to secretory capacity by processing the XBP-1 mRNA Nature 2002 415 92 96 10.1038/415092a 11780124

[B29] Lee AH Iwakoshi NN Glimcher LH XBP-1 regulates a subset of endoplasmic reticulum resident chaperone genes in the unfolded protein response Mol Cell Biol 2003 23 7448 7459 10.1128/MCB.23.21.7448-7459.2003 14559994 PMC207643

[B30] Ohoka N Yoshii S Hattori T Onozaki K Hayashi H TRB3, a novel ER stress-inducible gene, is induced via ATF4-CHOP pathway and is involved in cell death EMBO J 2005 24 1243 1255 10.1038/sj.emboj.7600596 15775988 PMC556400

[B31] Okada T Yoshida H Akazawa R Negishi M Mori K Distinct roles of ATF6 and PERK in transcription during the mammalian unfolded protein response Biochem J 2002 366 585 594 10.1042/BJ20020391 12014989 PMC1222788

[B32] Sun GD Kobayashi T Abe M Tada N Adachi H Shiota A Totsuka Y Hino O The endoplasmic reticulum stress-inducible protein Niban regulates eIF2alpha and S6K1/4E-BP1 phosphorylation Biochem Biophys Res Commun 2007 360 181 187 10.1016/j.bbrc.2007.06.021 17588536

[B33] Kimball SR Do AN Kutzler L Cavener DR Jefferson LS Rapid turnover of the mTOR complex 1 (mTORC1) repressor REDD1 and activation of mTORC1 signalling following inhibition of protein synthesis J Biol Chem 2008 283 3465 3475 10.1074/jbc.M706643200 18070882 PMC2654224

[B34] Geng J Klionsky DJ The Atg8 and Atg12 ubiquitin-like conjugation systems in macroautophagy EMBO Rep 2008 9 859 864 10.1038/embor.2008.163 18704115 PMC2529362

[B35] Proikas-Cezanne T Waddell S Gaugel A Frickey T Lupas A Nordheim A WIPI-1alpha (WIPI49), a member of the novel 7-bladed WIPI protein family, is aberrantly expressed in human cancer and is linked to starvation-induced autophagy Oncogene 2004 23 9314 9325 10.1038/sj.onc.1208331 15602573

[B36] Sato H Tamba M Ishii T Bannai S Cloning and expression of a plasma membrane cystine/glutamate exchange transporter composed of two distinct proteins J Biol Chem 274 11455 11458 10.1074/jbc.274.17.11455 10206947

[B37] Nicklin P Bergman P Zhang B Triantafellow E Wang H Nyfeler B Yang H Hild M Kung C Wilson C Myer VE MacKeigan JP Porter JA Wang YK Cantley LC Finan PM Murphy LO Bidirectional transport of amino acids regulates mTOR and autophagy Cell 2009 136 521 534 10.1016/j.cell.2008.11.044 19203585 PMC3733119

[B38] Gourley GR Bilirubin metabolism and kernicterus Adv Pediatr 1997 44 173 229 9265971

[B39] Zucker SD Goessling W Hoppin AG Unconjugated bilirubin exhibits spontaneous diffusion through model lipid bilayers and native hepatocyte membranes J Biol Chem 1999 274 10852 10862 10.1074/jbc.274.16.10852 10196162

[B40] Rodrigues CM Sola S Brites D Bilirubin induces apoptosis via the mitochondrial pathway in developing rat brain neurons Hepatology 2002 35 1186 1195 10.1053/jhep.2002.32967 11981769

[B41] Gennuso F Fernetti C Tirolo C Testa N L'Episcopo F Caniglia S Morale MC Ostrow JD Pascolo L Tiribelli C Marchetti B Bilirubin protects astrocytes from its own toxicity by inducing up-regulation and translocation of multidrug resistance-associated protein 1 (Mrp1) Proc Natl Acad Sci USA 2004 101 2470 2475 10.1073/pnas.0308452100 14983033 PMC356974

[B42] Ostrow JD Pascolo L Brites D Tiribelli C Molecular basis of bilirubin-induced neurotoxicity Trends Mol Med 2004 10 65 70 10.1016/j.molmed.2003.12.003 15102359

[B43] Oakes GH Bend JR Early steps in bilirubin-mediated apoptosis in murine hepatoma (Hepa 1c1c7) cells are characterized by aryl hydrocarbon receptor-independent oxidative stress and activation of the mitochondrial pathway J Biochem Mol Toxicol 2005 19 244 55 10.1002/jbt.20086 16173058

[B44] Vitek L Schwertner HA The heme catabolic pathway and its protective effects on oxidative stress-mediated diseases Adv Clin Chem 2007 43 1 57 full_text 17249379 10.1016/s0065-2423(06)43001-8

[B45] Kumar S Guha M Choubey V Maity P Srivastava K Puri SK Bandyopadhyay U Bilirubin inhibits Plasmodium falciparum growth through the generation of reactive oxygen species Free Radic Biol Med 2008 44 602 613 10.1016/j.freeradbiomed.2007.10.057 18070610

[B46] Seubert JM Darmon AJ El-Kadi AO D'Souza SJ Bend JR Apoptosis in murine hepatoma hepa 1c1c7 wild-type, C12, and C4 cells mediated by bilirubin Mol Pharmacol 2002 62 257 264 10.1124/mol.62.2.257 12130676

[B47] Cesaratto L Calligaris SD Vascotto C Deganuto M Bellarosa C Quadrifoglio F Ostrow JD Tiribelli C Tell G Bilirubin-induced cell toxicity involves PTEN activation through an APE1/Ref-1-dependent pathway J Mol Med 2007 85 1099 112 10.1007/s00109-007-0204-3 17479230

[B48] Ollinger R Bilban M Erat A Froio A McDaid J Tyagi S Csizmadia E Graça-Souza AV Liloia A Soares MP Otterbein LE Usheva A Yamashita K Bach FH Bilirubin: a natural inhibitor of vascular smooth muscle cell proliferation Circulation 2005 112 1030 1039 10.1161/CIRCULATIONAHA.104.528802 16087796

[B49] Ollinger R Kogler P Troppmair J Hermann M Wurm M Drasche A Königsrainer I Amberger A Weiss H Ofner D Bach FH Margreiter Bilirubin inhibits tumor cell growth via activation of ERK Cell Cycle 2007 6 3078 3085 18073533 10.4161/cc.6.24.5022

[B50] Nguyen N Bonzo JA Chen S Chouinard S Kelner MJ Hardiman G Bélanger A Tukey RH Disruption of the ugt1 locus in mice resembles human Crigler-Najjar type I disease J Biol Chem 2008 283 7901 7911 10.1074/jbc.M709244200 18180294

[B51] Silva RF Rodrigues CM Brites D Bilirubin-induced apoptosis in cultured rat neural cells is aggravated by chenodeoxycholic acid but prevented by ursodeoxycholic acid J Hepatol 2001 34 402 408 10.1016/S0168-8278(01)00015-0 11322201

[B52] Han Z Hu P Ni D Bilirubin induced apoptosis of human neuroblastoma cell line SH-SY5Y and affected the mitochondrial membrane potential Zhonghua Er Bi Yan Hou Ke Za Zhi 2002 37 243 246 12772405

[B53] Paschen W Mengesdorf T Endoplasmic reticulum stress response and neurodegeneration Cell Calcium 2005 38 409 415 10.1016/j.ceca.2005.06.019 16087231

[B54] Vembar SS Brodsky JL One step at a time: endoplasmic reticulum-associated degradation Nat Rev Mol Cell Biol 2008 9 944 957 10.1038/nrm2546 19002207 PMC2654601

[B55] Matus S Lisbona F Torres M León C Thielen P Hetz C The stress rheostat: an interplay between the unfolded protein response (UPR) and autophagy in neurodegeneration Curr Mol Med 2008 8 157 172 10.2174/156652408784221324 18473817

[B56] Rubinsztein DC The roles of intracellular protein-degradation pathways in neurodegeneration Nature 2006 443 780 786 10.1038/nature05291 17051204

[B57] Sedlak TW Saleh M Higginson DS Paul BD Juluri KR Snyder SH Bilirubin and glutathione have complementary antioxidant and cytoprotective roles Proc Natl Acad Sci USA 2009 106 5171 5176 10.1073/pnas.0813132106 19286972 PMC2664041

[B58] Savaskan NE Heckel A Hahnen E Engelhorn T Doerfler A Ganslandt O Nimsky C Buchfelder M Eyüpoglu IY Small interfering RNA-mediated xCT silencing in gliomas inhibits neurodegeneration and alleviates brain edema Nat Med 2008 14 629 632 10.1038/nm1772 18469825

[B59] McDonagh AF Assisi F The ready isomerization of bilirubin IX- in aqueous solution Biochem J 1972 129 797 800 4659001 10.1042/bj1290797 PMC1174183

[B60] Roca L Calligaris S Wennberg RP Ahlfors CE Malik SG Ostrow JD Tiribelli C Factors affecting the binding of bilirubin to serum albumins: validation and application of the peroxidase method Pediatr Res 2006 60 724 728 10.1203/01.pdr.0000245992.89965.94 17065581

[B61] Denizot F Lang R Rapid colorimetric assay for cell growth and survival. Modifications to the tetrazolium dye procedure giving improved sensitivity and reliability J Immunol Methods 1986 89 271 277 10.1016/0022-1759(86)90368-6 3486233

[B62] Huber W Von Heydebreck A Sültmann H Poustka A Vingron M Variance stabilization applied to microarray data calibration and to the quantification of differential expression Bioinformatics 2002 18 S96 S104 12169536 10.1093/bioinformatics/18.suppl_1.s96

[B63] McClintick JN Edenberg HJ Effects of filtering by Present call on analysis of microarray experiments BMC Bioinformatics 2006 7 49 10.1186/1471-2105-7-49 16448562 PMC1409797

[B64] Saeed AI Sharov V White J Li J Liang W Bhagabati N Braisted J Klapa M Currier T Thiagarajan M Sturn A Snuffin M Rezantsev A Popov D Ryltsov A Kostukovich E Borisovsky I Liu Z Vinsavich A Trush V Quackenbush J TM4: a free, open-source system for microarray data management and analysis Biotechniques 2003 34 374 378 12613259 10.2144/03342mt01

[B65] Tusher VG Tibshirani R Chu G Significance analysis of microarrays applied to the ionizing radiation response Proc Natl Acad Sci USA 2001 98 5116 5121 10.1073/pnas.091062498 11309499 PMC33173

[B66] Chu VT Gottardo R Raftery AE Bumgarner RE Yeung KY MeV+R: using MeV as a graphical user interface for Bioconductor applications in microarray analysis Genome Biol 2008 9 R118 10.1186/gb-2008-9-7-r118 18652698 PMC2530872

[B67] Ashburner M Ball CA Blake JA Botstein D Butler H Cherry JM Davis AP Dolinski K Dwight SS Eppig JT Harris MA Hill DP Issel-Tarver L Kasarskis A Lewis S Matese JC Richardson JE Ringwald M Rubin GM Sherlock G Gene ontology: tool for the unification of biology. The Gene Ontology Consortium Nat Genet 2000 25 25 29 10.1038/75556 10802651 PMC3037419

